# Simulating relational event history data: why and how

**DOI:** 10.1007/s42001-025-00427-2

**Published:** 2025-08-21

**Authors:** Rumana Lakdawala, Joris Mulder, Roger Leenders

**Affiliations:** 1 Department of Methodology and Statistics, Tilburg School of Social and Behavioral Sciences, Tilburg University, Tilburg, The Netherlands; 2 Jheronimus Academy of Data Science, ‘s-Hertogenbosch, The Netherlands; 3 Department of Organization Studies, Tilburg School of Social and Behavioral Sciences, Tilburg University, Tilburg, The Netherlands

**Keywords:** Relational events, Simulation techniques, Temporal social networks, Model fit assessment, Interventions, Actor-oriented models, Dyadic interaction models

## Abstract

Many important social phenomena are characterized by repeated interactions among individuals over time such as email exchanges in an organization or face-to-face interactions in a classroom. To understand the underlying mechanisms of social interaction dynamics, statistical simulation techniques for network data at fine temporal granularity are crucial. This article makes two contributions to the field. First, we present statistical frameworks to simulate relational event networks under dyadic and actor-oriented relational event models implemented in an R package remulate. Second, we show how this simulation framework can address key challenges in temporal social network analysis through five case studies. The first study illustrates the necessity of simulation based techniques for model assessment, using a network of criminal gangs. The second shows how simulation supports social theory development which is illustrated via optimal distinctiveness theory. The third explores simulation for understanding the effects of network interventions. In the fourth study, we illustrate how simulation-based analysis can be used to assess the sensitivity of relational event models. The fifth study demonstrates how simulation frameworks can be used to make predictions about future relational dynamics. Through these case studies and software, researchers will be able to better understand social interaction dynamics using relational event data from real-life networks.

## Introduction

Our understanding of social interaction mechanisms between actors has been enhanced by dynamic network-based approaches. These actors can represent individuals, groups of individuals, organizations, or even countries. A network approach allows for the representation of actors by vertices and an event or interaction between the actors by the edges between the vertices. In traditional, static network analysis, edges are assumed to be stable, and their presence or absence is indicative for the state the network is in. In continuous-time dynamic network analysis (which is the focus of this paper), edges can appear and/or dissolve in real time at any time. To distinguish between edges that are stable and edges that can be in constant flux, the latter are often termed “relational events.” The analysis of relational event data has the potential for a deeper understanding of the underlying social structures and how these evolve over time. This allows for the investigation of relationships between actors, dyads, and collective behaviour in the network. Particularly, the temporal information yields insight into how variation in individual-level dynamics affects network-level characteristics, which in turn further affect individual behaviour. Several statistical methods have been developed to study dynamic relational event history data for longitudinal social network analysis [26, 27, 32, 43, 46]. Of particular interest to us is the relational event model (REM) proposed by [12, 48], which allow dynamic social network analyses when the exact time-stamp of the events are available. In these relational event models, the event rate between actors is modelled as a function of the actors’ past history and exogenous information available about the actors (nodal covariates) or pairs of actors (dyadic covariates) in the network. These models provide a flexible framework for network inference that allows for the estimation of various drivers of social interaction behaviour, such as homophily, reciprocity, transitivity, and preferential attachment.

Despite its usefulness to quantify the relative importance of network drivers of social evolution, truly understanding the underlying social mechanisms between actors based on relational event history data remains an enormous challenge. For example, different drivers of interaction in a network might yield similar observed patterns of social interaction behaviour, making it difficult to disentangle the true underlying processes. This is echoed in the famous quote [8] that “all models are wrong...”. Simulation techniques have the potential to tackle these important current challenges in temporal social network modelling due to their ability to assess the impact of different model specifications on the generated network’s characteristics in a direct manner. To give an example, Juozaitienė and Wit [29] showed that not accounting for nodal heterogeneity can lead to “ghost” triadic effects.

For this reason, the aims of the current paper are twofold. First, it further develops and extends simulation techniques under flexible relational event modelling frameworks. Simulation frameworks are proposed for dyadic relational event models [12] and actor-oriented relational event models [49]. Unlike existing approaches, the proposed simulators support time-varying network effects, constrained risk sets, and various memory decay function to compute endogenous statistics allowing the study of the elapsed time of past events on the event rate in the (near) future. Moreover, the simulation software is considerably faster which will be useful to statistical practice. Second, the paper explores the potential of simulation techniques to improve social network analyses to better understand social interaction behaviour in real-life applications. This is achieved by meeting the following objectives:

1. Assess goodness of fit: For a richer goodness of fit assessment, key network characteristics of the empirical relational event networks (such as degree distributions, density, triadic sub-structures, inter-event time distribution) can be compared to the simulated networks under the fitted model.
2. Evaluate network interventions: Relational event simulations can be used to evaluate network interventions by investigating the temporal dynamics of social networks under various intervention scenarios. This can be achieved by varying network characteristics and evaluating their impact on network dynamics. For example, researchers can use simulations to explore how quickly networks respond to interventions, how long interventions must be performed to achieve desired outcomes, and whether the effects of interventions persist over time or fade away. Additionally, simulations can be used to study the effects of targeting specific actors in the network and to identify the most effective intervention strategies for different network configurations.
3. Develop theory: Relational event simulations can be a powerful tool for developing social theories by providing a way to test and refine theoretical models in a controlled and systematic way. Simulations can be used to validate, test, and extend existing theories as well as developing new theories about the social phenomena that drive the interactions in social networks. In addition, simulations can help identify gaps in existing theories or data. For example, if a simulated network does not match the observed network characteristics, this could indicate that the theoretical model is incomplete or that data collection methods need to be revised. By identifying these gaps, researchers can develop more comprehensive theories and improve data collection methods.
4. Make predictions: Relational event simulations fitted with predictive models can help us understand how relational sequences may evolve beyond the observation period, enabling us to make predictions about the most likely events in the near future or explore alternate scenarios for how network dynamics may develop over a longer period.
5. Evaluate the sensitivity and statistical power of the model: Simulation-based analysis can be used to assess the sensitivity of relational event models under specific scenarios or when assumptions are violated. Researchers can also study the power, accuracy, and precision of relational event analyses [e.g., 44] in idealized circumstances to test and benchmark novel extensions of the model where the true conditions that generated the data are known. Furthermore, various network characteristics (i.e. network size, combinations of endogenous and (or) exogenous effects, interaction effects, scaling of covariates, etc.) can be varied to evaluate the impact of varied conditions on the robustness, power, and stability of model estimates.

Generating relational event sequences is not a simple task however. For the standard REM, assuming a constant risk set, time-invariant parameters, writing code to generate event sequences is in itself straightforward, but doing it in a way that is efficient (in terms of computation time and memory usage) requires quite efficient code in order to make generating sequences with a fair number of actors and events practically feasible (see Table 1 for an example). Generating event sequences under varying risk sets, time-varying parameters, with memory decay, or other complexities that researchers are often interested in is far from trivial, especially if it needs to be done in a manner that is reproducible, transparent, extendable, and efficient. The tools we present in this paper can reliably perform such tasks, such that the researcher can focus on the five uses we outlined above.

This article is organized as follows. In Sect. 2, we introduce the dyadic and actor-oriented relational event models and outline the general-purpose simulation frameworks for these models that can be customized in various ways using problem-specific information. Next, we illustrate a range of simulation scenarios that showcase the broad potential of the simulation frameworks in an attempt to inspire and guide new research. In Sect. 3 we demonstrate how the simulations can be used to assess goodness of fit of relational event models using a criminal network of attacks amongst gangs. In Sect. 4, we describe how simulations can be used to develop theories about social phenomena that drive interactions and provide an example by simulating a well-known social theory and testing a boundary condition on this theory using simulations. In Sect. 5, we discuss how the simulation framework can be used to investigate the outcomes of network-based interventions. We demonstrate how strategies for network-based interventions can be simulated and compared in an organizational network. In Sect. 6, we discuss how simulations can be utilized to evaluate the sensitivity of relational event models with an example of time-varying network effects. In Sect. 7, we illustrate using the criminal gangs dataset, how simulation frameworks can be used to make predictions about future relational dynamics. Finally, we conclude with a discussion on the limitations and future prospects of simulation techniques in Sect. 8.

## Simulating relational event networks

A relational event can be thought of as an event in which a sending actor (e.g. a person, group of individuals or other entity) directs an action to a receiving actor (e.g. another person, group, organisation, etc.) in the form of a discrete instantaneous event at a certain time point. The specification for an observed event $e = (i,j,t)$ entails a sender $i \in \mathcal {A}$, a receiver $j \in \mathcal {A}$, and *t*, the time at which the event was observed, where $\mathcal {A}$ is a set of actors.^1^ An event represents one step in the dynamic network, and a temporal sequence of relational events forms the relational event history [14]. In this paper, we describe networks in which the connections are directed (that is, an event from $i \rightarrow j$ is distinct from $j \rightarrow i$) and does not contain self-loops (that is, from $i \rightarrow i$). However, extensions to undirected ties are straightforward.

In order to simulate a relational event network sequence^2^*E* in a time window $[0, \tau )$, every event *e* in the sequence *E* should be specified. This involves generating the event times $t \in [0,\tau )$ at which each event occurs and the dyad (*i*, *j*) associated with each event. This process of generating event sequences can be described under the dyadic Relational Event Model (REM) of [12] as well as the actor-oriented Dynamic Network Actor Model (DyNAM) of [49]. We describe each of these simulation methods in detail in the following sections.

### Simulation framework 1: relational event model (REM)

Consider a sequence of *M* events $E = \{ e_1, e_2, \dots e_M\}$ such that. $t_m < t_{m+1}$. An event $e_m = (i_m,j_m,t_m)$, entails a sender $i_m \in \mathcal {A}$, a receiver $j_m \in \mathcal {A}$, and the time at which the event was observed $t_m$. The set of possible events that can occur at any time is called the risk set $\mathcal {R} \subseteq \{ (i,j): i,j \in \mathcal {A}\}$. The risk set may be constrained so that certain actors cannot interact with each other. Therefore, the constrained riskset can be fixed or vary exogenously, where the possibility of occurrence of certain events is determined by exogenous factors such as availability of actors, location, or other circumstances specific to the research setting.

In REM, each dyad has its own rate of occurrence. A higher rate implies that the event involving that dyad is more likely to occur soon, and a lower rate implies that the occurrence of the event is more rare. The rate is modelled as a log-linear function of the endogenous and exogenous statistics pertaining to that dyad along with the parameters $\varvec{\beta }(t)$ that represent the strength of these statistics to explain social interaction behaviour in the network. The occurrence of events is modelled using a piece-wise constant hazard model. Under the piece-wise constant model, the rates are assumed to only change when an event occurs (anywhere in the network). Thus, as time passes and events are observed, the rate is updated to reflect the new network history. The rate $\lambda ^{dyadic}_{ij}$ of a dyad (*i*, *j*) at time *t* is then specified in log-linear form as:

1 $$\begin{aligned} \lambda ^{dyad}_{ij}(t ) = {\left\{ \begin{array}{ll} \text {exp}\{ \varvec{\beta }(t)^T \; X(i,j,E_{t}) \} & \ (i,j) \in \mathcal {R} \\ 0 & \ (i,j) \notin \mathcal {R} \end{array}\right. } \end{aligned}$$

where $X(i,j,E_{t})$ is a vector of *P* statistics for the dyad (*i*, *j*) in the sequence of events $E_t$ until time t and $\varvec{\beta }(t) \in \mathrm{I\!R^P}$ is the vector of corresponding parameters at time *t* (the parameters may be constant and do not vary over time, in which case $\beta$ is a constant. The statistics vector $X(i,j,E_{t})$ can capture either endogenous network characteristics as a function of past events in the history $E_{t}$ before time *t* or the exogenous actor or dyadic attributes. The notation for rate has a superscript ‘dyad’ to distinguish it from the rate under the actor-oriented model introduced in the next section.

Under the piece-wise constant hazard model, the waiting time $\delta _m = t_{m} - t_{m-1}$ between subsequent events are assumed to be conditionally independent and are specified by an exponential distribution, i.e.,

2 $$\begin{aligned} p^{dyad}(\delta _m|E_{t_{m-1}},\varvec{\beta }(t_{m-1}),X) \sim \text {Exp}(\Lambda ^{dyad}(t_{m-1} )) \end{aligned}$$

with the cumulative rate as distributional parameter, given by

$$\begin{aligned} \Lambda ^{dyad}(t_{m-1}) = \sum \limits _{(i,j) \in \mathcal {R} } \lambda _{ij}^{dyad}(t_{m-1}). \end{aligned}$$

The probability that dyad $(i,j) \in \mathcal {R}$ is involved in the next observed event at time *t*, follows a multinomial distribution where the probabilities are proportional to the dyadic rates:

3 $$\begin{aligned} {\begin{matrix} p^{dyad}(i,j \mid t , \varvec{\beta }(t), X, E_{t}) = \frac{ \lambda ^{dyad}_{ij}(t ) }{ \Lambda ^{dyad}(t ) }. \; \end{matrix}} \end{aligned}$$

In order to simulate relational event networks, the user needs to specify which endogenous or exogenous network statistics [e.g., 35] are included in the model and the magnitude of the corresponding parameters (based on theory, a specific dynamic of theoretical interest or a fitted model). If preferred, it is also possible to initialize simulations with a predefined starting sequence $E_0$. This is useful if a user wants to predict the immediate future of an empirical network or to simulate using different models from the same event history. The inputs to the simulation algorithm play an important role in determining the characteristics of the sequence output. The number of actors *N* in the network determines the size of the risk set as $N(N-1)$ (when all dyads are at risk) and $\tau$, the time until the simulation algorithm is run determines the number of events in the sequence. The parameters $\varvec{\beta }(t)$ capture the sign and magnitude of the effect of the statistics. Given these inputs, a relational sequence can be simulated using Algorithm 1.

**Algorithm 1:**
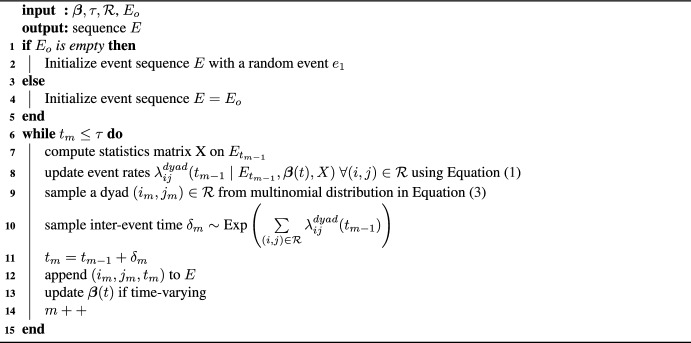
Simulation of a relational event sequence under dyadic model

### Simulation framework 2: dynamic network actor model (DyNAM)

Instead of all dyads competing with each other to participate in the next event, under the DyNAM the actors compete with each other to become the next sender, where each actor has its own rate parameter to be the next sender. A higher rate implies that the actor is more likely to be the sender of the next event. The rate of the actor as a sender is specified similarly to the rate parameter in a dyadic REM using a log-linear model of endogenous and exogenous sender statistics, which are now summarized in the statistics matrix $X^s$. The time of the next event then follows an exponential distribution,

$$\begin{aligned} p^{actor}(\delta _m|E_{t},\varvec{\gamma }(t),X^s)\sim \text {Exp}(\Lambda ^{sender}(t)), \end{aligned}$$

where $\Lambda ^{sender}(t) = \sum \limits _{i \in A } \lambda _{i}^{sender}(t)$, with $\mathcal {R}^s$ denotes the set of actors that are at risk to become a sender, and the rate parameter of actor *i* to become a sender is defined by

4 $$\begin{aligned} \lambda _{i}^{sender}(t) = \exp \{ \varvec{\gamma }(t)^TX^s(i,E_t), \} \end{aligned}$$

where $\gamma$ denotes the vector of coefficients that quantify the importance of the respective statistic in $X^s$. Furthermore, the probability that actor *i* will become a sender is proportional to the respective rate parameters according to a multinomial distribution.

5 $$\begin{aligned} p^{actor}(i \mid E_{t} , \varvec{\gamma }(t),X^s) = \frac{\lambda _{i}^{sender}(t) }{ \Lambda ^{sender}(t). } \end{aligned}$$

Finally, the probability of actor *j* to become the receiver is modeled conditionally on the sender *i* using a multinomial distribution with probability

6 $$\begin{aligned} p^{actor}(j | i , t, E_{t} , \varvec{\alpha }(t),X^r ) = \frac{\lambda _{j|i}^{receiver}(t) }{ \Lambda _{i}^{receiver}(t) } \end{aligned}$$

where the preference parameter of actor *j* to become the receiver is given by

$$\begin{aligned} \lambda _{j|i}^{receiver}(t)= \text {exp}(\varvec{\alpha }(t)^T X^r(i,j,E_{t} ), \end{aligned}$$

which is modelled in a similar manner as the rate parameter, and the summation is given by

$$\begin{aligned} \Lambda _i^{receiver}(t)= \sum _{j \in A \backslash \{i\}} \lambda _{j|i}^{receiver}(t). \end{aligned}$$

The simulation framework of relational event sequences under DyNAM is then summarized in Algorithm 2

**Algorithm 2:**
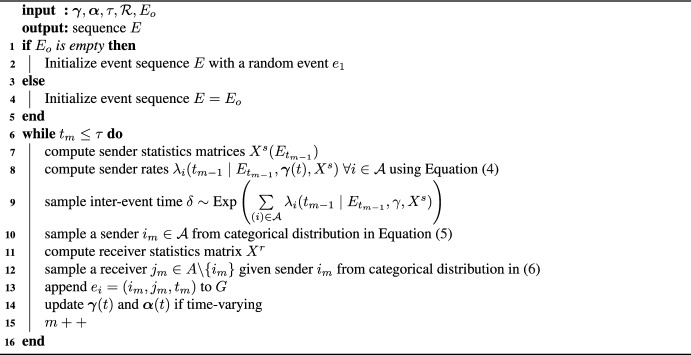
Simulation of relational event sequence under actor-oriented model

The main differences between the dyadic relational event model and its actor-oriented counterpart (i.e., the DyNAM) can be understood from how they model the occurrence of relational events. In the dyadic REM, the timing of the next event and the dyad that is observed are modelled using dyad-specific rate parameters. Alternatively, in the actor-oriented model, the timing and the sender of the next event are modelled via actor (sender) specific rate parameters, and the next receiver is modelled conditionally on the sender (i.e., *chosen* by the sender) where all actors have separate rate parameters as potential receivers [49]. These differences in modelling are also reflected in the simulation frameworks. In the dyadic model, all dyads are competing to be sampled as the next observed event, whereas in the actor-oriented model, the actors first compete to become the sender, and then the remaining actors compete to become the receiver that is chosen by the given sender. The two models also differ in how they conceive of network change. In the dyadic model, the building block is the dyad, and the model assumes the two participating actors in the dyad to actively determine whether and when they will interact. In the actor-oriented model, on the other hand, the agency is at the level of the sender, who determines when to get active. Therefore, the latter model is conceptually actor driven rather than dyad driven. In the simulation frameworks, this difference translates to which statistics are used to determine the next sampled event. In case of the dyadic REM, dyadic statistics are used to sample the next event, whereas in case of actor-oriented model, actor-focused statistics are used to sample the sender of the next event and dyadic statistics are used to make the choice of the receiver given the sender.

### remulate: R package to simulate relational event histories

This article introduces the R package remulate [34]. The package is developed to help researchers simulate relational event histories. The package aims to reduce the complexity associated with simulation techniques and to make them more accessible to researchers in social networks. The package enables the user to simulate using a wide range of commonly used exogenous and endogenous statistics for the tie- and actor-oriented relational event model approaches, as well as several important extensions.

Unlike relevent [13] which also allows users to simulate relational event histories, remulate has the following additional features:

1. Support for dyadic relational event models [12] as well as actor-oriented relational event models [49].
2. Support of a larger collection of precomputed endogenous statistics with various methods of normalization and standardization and support for interaction terms.
3. Support for different types of memory decay functions [such as exponential or stepwise decay, 4, 5, 10] to support studying the influence of the transpired time since past events occurred on the future event rate between actors.
4. Support for constrained risk sets (i.e. if two actors cannot interact, events involving that dyad is not sampled).
5. Support for time-varying network parameters to study instantaneous, gradual, and periodic changes in drivers of social interaction [38, 40, 45].
6. Support for frailty relational event models [to capture nodal heterogeneity, e.g., see 30, 39].
7. Support for relational event block models [17].

**Fig. 1:**
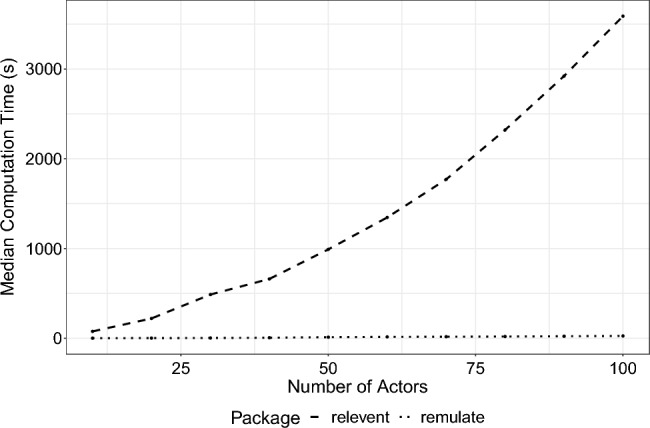
Median computation (s) time for simulating 25 identical relational event sequences of 1000 events for varying number of actors using R packages: remulate and relevent

Furthermore, we compared the efficiency of remulate and relevent in simulating relational event sequences using inertia and reciprocity effects. Both methods generated 1000 events while varying the number of actors between $\{10, 20, 30, 40, 50, 60, 70, 80, 90, 100\}$. The median runtime^3^ (in s) over 25 runs for each actor count is presented in Figure 1 and Table 1. The results show that remulate is substantially faster than relevent, which is particularly important for simulations, as most applications require generating a large number of networks.

**Table 1:** Median computation time (s) for simulating 25 identical relational event sequences of 1000 events using R packages: remulate and relevent

Actors	remulate	relevent
10	1.07	76.5
20	1.81	220.5
30	3.1	486.9
40	5.8	660.9
50	12.0	990.4
60	15.6	1346.1
70	17.4	1770.1
80	19.8	2320.9
90	22.5	2921.2
100	25.9	3588.7

Appendix 2 presents exemplary R code on how to simulate from tie-oriented and actor-oriented relational event models using the remulate package.

## Assessing goodness-of-fit through simulations

In the following we will present extensive applications that highlight why relational event simulators are essential for temporal social network research. We begin with assessing the goodness of model fit through simulations.

Typical approaches to evaluating goodness-of-fit of relational event models involve a comparison of different models by balancing parsimony and accuracy. The preferred model generally contains the fewest parameters while yielding a satisfactory fit for the observed relational events. This usually involves using information criteria such as the Akaike Information Criterion (AIC), the Bayesian Information Criterion (BIC), or other likelihood-based goodness-of-fit measures. Although these criteria are useful as a relative measure of fit when comparing models, they do not give an indication of the model fitness in an absolute sense nor do they provide a substantive direction of possible misfit. Thus, when comparing multiple relational event models using such general information criteria, the “best” model may still have a very poor fit to the observed event sequence in terms of capturing important network characteristics and may result in poor predictions.

On the other hand, simulation-based methods can be used to assess model fit in an absolute sense by assessing whether important network characteristics in the data are also present in the simulated data using the fitted model. If simulated sequences based on a fitted model bear little resemblance to the observed sequence (or miss important characteristics of the network dynamic), this generally suggests a poor fit of the model to the data. Misfit can occur due to the inclusion of effects that are not operational in the data or due to exclusion of key effects or external factors that play an important role in the network dynamics. Inferences based on misfitted models are therefore (theoretically) unreliable, and simulation-based methods are needed to assess whether the model results in a reasonable fit to the observed data sequence before drawing inferences.

Before describing the general methodology, we briefly mention some previous work on simulation-based methods for model assessment in social network research. Hunter et al. [28] introduced simulation-based goodness-of-fit tests for social networks in exponential random graph models (ERGMs) using structural indices such as degree distributions, edge-wise shared partners, and geodesic distance distributions. Snijders and Steglich [47] introduce a variety of structural fit indices to describe cross-sectional data such as the size of the largest component, the number of components, the median geodesic distance, the transitivity coefficient, the variance of the in or out degree divided by the mean, the correlation between the in and out degrees, the graph hierarchy, and the least upperboundedness. Lospinoso and Snijders [37] include higher order indices in the form of triad census to assess if the simulated networks accurately resemble triadic structures in the observed network. Wang et al. [53] describe behavioural indices to assess fit for models that jointly model behaviour and network structure. Brandenberger [9] considered the accuracy of event predictions to assess the goodness-of-fit of relational event models for political networks. Because relational event models not only model the network structure but also the timing and order of events, goodness-of-fit tests would be incomplete without assessing the fit of candidate models on temporal indices. It is possible that the model fits the data well within certain time periods but not in others or that events between certain actors in the predicted dataset are farther apart than in the observed network. In addition, it is possible to compute structural indices that respect the timing and order of events in relational event datasets, such as temporal degree, temporal reachability, and temporal betweenness [20]. Nicosia et al. [42] similarly define temporal connectedness, various temporal centrality indices, and temporal shortest paths for time-respecting paths in dynamic networks. These temporal indices enhance the fit indices used for static or longitudinal networks, by also incorporating the timing information available from relational event datasets. In addition [2] suggest examining internal time structures in relational event models to capture the specific sequence and timing of events, such as reciprocity and transitive closure, to ensure that the model accurately reflects the temporal patterns observed in social interactions.

The indices mentioned above are just a few examples from the literature on social networks to assess the goodness of fit. In fact, the number of temporal network characteristics to assess is unlimited. Moreover, when assessing goodness of fit, we believe that researchers should focus on reproducing specific aspects of the data. that are of theoretical and/or practical interest rather than applying a default set of statistics. Ultimately, the choice of the fit indices should depend on the specific phenomena the researchers intend to address in their analysis. Therefore, it is impossible to provide an exhaustive set of network characteristics for general use in relational event analysis.

In the context of relational event models, this approach to evaluating goodness-of-fit (i) allows researchers to test the model’s fit across a set of interpretable structural and social indices and (ii) guides the selection of network effects to be included in the model by pointing the researcher towards a direction in which the model may be misfit or lacking in specification.

### Assessing goodness-of-fit of a model for violent interactions between criminal gangs

To demonstrate the evaluation of goodness-of-fit through simulations, we evaluate the fit of a model presented in a recent study by [24]. The study focuses on the dynamics of intergang violence in Los Angeles using a relational event modelling approach. The relational event history comprises of violent incidents involving 33 gangs spanning over three years. The authors examine various mechanisms that drive the continuation of violence in this network. The authors focus on how gang characteristics, spatial proximity, enduring rivalries, and how the dynamics of past conflicts influence future violent events. The authors argue that while retaliation is a significant mechanism, other factors such as established rivalries and inertia play crucial roles in driving future violence. The authors of the article attempt to assess the goodness-of-fit of their model by checking how well the fitted model can predict the observed events in-sample: For every observed event, the likelihood that it would be observed was checked given the predictor variables at that point. Although this analysis indicated that the fitted model has a high predictive accuracy, this assessment is unable to answer whether the entire sequence of relational events is plausible under the fitted model when it would be used to generate entire sequences of events “from scratch”. That is the purpose of the current simulation analysis. We investigate the fit of various data characteristics on gang violence including gang size, inter-dyadic distance, geodesic hop-path lengths, degree statistics, and interevent times between rival gangs.

**Fig. 2:**
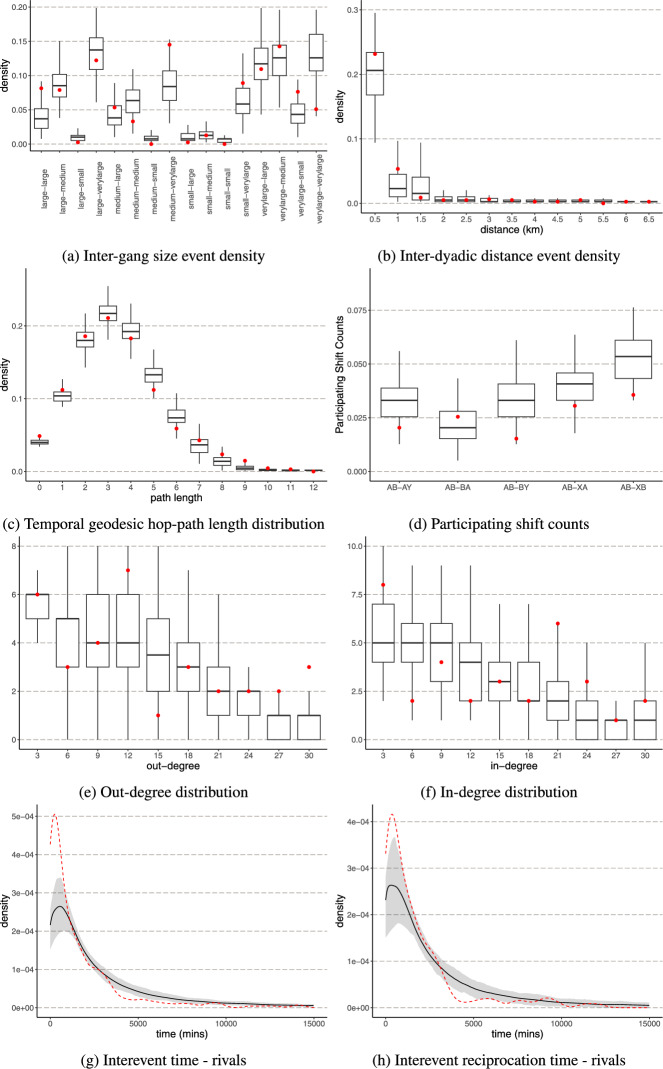
Goodness-of-fit comparison. Each figure plots the values of the fit indices over 100 simulated sequences and the observed data. In figures **a**–**f** the red crosses represent the observed data and the boxplots depict the distribution of the fit indices across simulated networks. In figures **g**–**h** the dotted red line represents the observed data

To assess the goodness of fit for the relational event models presented in this study, we simulate from the fitted relational event models (see Appendix 4 for the R-code) using the maximum likelihood estimates presented in Table 2 of [24], who also provide a detailed description of all network statistics. The plots in Fig. 2 illustrate the goodness-of-fit for the model, comparing the characteristics of simulated networks with the actual data observed in the context of street gang violence. Each subplot highlights a different characteristic of the network derived from violent conflicts between gangs. Figure 2a shows the density of events categorized by the size of the participating gangs in the dyads. In the context of the paper, understanding these interactions is crucial because larger gangs might have different resources, territorial ambitions, and levels of influence, which can affect their interaction patterns with smaller gangs. The observed data (red crosses) are relatively well aligned with the median of the simulated distributions, implying that the model captures the frequency of events across gang size effectively. There is a slight misfit involving dyads with a larger gang size, which might necessitate the need for an additional variable to effectively reproduce the interaction patterns of larger sized gangs. In Fig. 2b the density of events is plotted against the geographic distance between the dyads. The article highlights the importance of spatial proximity in the dynamics of gang violence, which makes it vital for the model to accurately simulate these interactions. The model appears to accurately capture the decline in event density as distance increases. The model slightly underestimates the frequency of localized conflicts that are more frequent and significant within closer geographical proximity. The Fig. 2c examines the distribution of temporal path lengths with shorter paths indicating more direct interactions over time between gangs (with lesser temporally respecting hops). The model reproduces the geodesic hop-path length distribution across the dyads in the network reasonably well. This fit index helps us to understand how violence spreads over time through the network, testing theories of violence contagion and diffusion within gang dynamics.

Participation shift counts of the participants of the dyadic turn taking events [21] are depicted in Fig. 2d. While, AB-BA and AB-BY type participating shifts were included in the model, the results suggest that the model is also able to reproduce the AB-AY, AB-XA and AB-XB turn taking events well. The out-degree distribution represents the number of conflicts initiated by each gang and is depicted in Fig. 2e. The observed data mostly align with the simulated medians for lower out-degrees but tend to fall outside the interquartile range for higher degrees. This could be due to the model not accounting for potential external factors that might influence a gang’s propensity for aggressive conflict initiation. In addition, we also look at the in-degree distribution of the data in Fig. 2f which suggest an adequate fit.

The last two Fig. 2g, h show the distribution of interevent events categorized by inertia and reciprocation among rival gangs. These timing-based indices test the model’s ability to quantify the rapidity and persistence of violence between rival gangs. It is vital for investigating how well the model replicates violence escalation and retaliation cycles, which are key aspects of gang dynamics described in the paper. In the figure, the observed data are represented by the red dotted line. Both plots demonstrate that conflicts between rival gangs are characterized by rapid sequences of events, with a significant number of actions occurring within a very short time frame following an initial event. However, the plot for reciprocation times between rival gangs has a somewhat less steep decline compared to the interevent times for rivals. This suggests that while immediate responses are common, there is slightly more variation in the timing of reciprocations, possibly due to differing strategic considerations or external factors influencing the timing of a gang’s response. The simulated results indicate an underestimation at very short inter-event times, suggesting the model might not fully capture the quickest escalations in gang conflict. Refining the model by including an interaction term between recent reciprocation and recent target persistence and rivalry might address the misfit. Additionally, short-term memory effects [10] that allow for more rapid changes in event probabilities immediately following a conflict event might allow the model to address the misfit.

Evaluating the goodness-of-fit using simulations has provided us with a more nuanced understanding of how well the model fits and highlighted potential areas of misfit compared to predictive performance or likelihood-based measures. Overall, while the model provides a good general fit to the observed data across several key indices, the detailed analysis provided insights into where the model falls short in capturing the dynamics of gang conflicts, such as the inter-event times between rival gangs. Understanding and refining these aspects of the model could lead to better predictions of gang conflict dynamics and more effective strategies for law enforcement and community interventions aimed at reducing the frequency and severity of these conflicts.

## Developing theories using simulations

The second important application of the relational event simulation techniques involves the development of social theories. Many social phenomena arise from repeated interactions among individuals in a social network over time. Undoubtedly, researchers continue to gain insight into the drivers of repeated social interactions through empirical studies. The relational event model itself has contributed greatly towards modelling the data arising out of such empirical research. Although this is useful for estimating the magnitude of network effects or confirming differences between different groups in specific empirical settings, fitted REMs are typically based on theoretical ideas of the researcher and contribute to testing existing theories rather than being employed as a way to develop or refine (new) theory. Simulations can provide a powerful and flexible tool for (further) developing or expanding theories [16] on why, how, and when interactions occur in a social network. The proposed relational event simulation frameworks provide flexibility in combining multiple network effects such as transitivity, homophily, or preferential attachment in one theoretical model, allowing researchers to test and develop richer theories about social interaction dynamics. Below we first describe how we can (further) develop social network theories using relational event simulations, and subsequently, we present an extensive application of the methodology to better understand group formation using optimal distinctive theory.

### Building, evaluating, and extending social network theories

Relational event simulations can be utilized to develop theories to (a) study the emergence of social phenomena, (b) evaluate the boundary conditions of theories, and (c) incorporate timing and dynamism in theories. We briefly elaborate on this below.

*Study the emergence of social phenomena* Emergence is a fundamental concept in complex systems theory [15] and social sciences and refers to the way in which collective phenomena arise from the interactions of individual entities [31]. Phenomena can be considered emergent when they cannot be reduced to the behaviour of individuals, but instead emerge from complex interactions among them. Relational event sequences can help explain elements of emergent properties that occur on a global level, but are a consequence of the history, timing, and underlying patterns of interpersonal event occurrence.

Simulations offer a powerful tool for studying the emergence of social phenomena [19, 22], especially those that are unexpected or counterintuitive. By simulating relational event models, researchers can generate scenarios that test the boundaries of existing theories and develop new insights into the mechanisms underlying emergent phenomena. In this sense, simulations offer a natural way of describing complex dynamics as a combination of familiar network mechanisms. Relational event simulation frameworks are particularly useful in studying emergent properties because they can capture the dynamic nature of social phenomena over time. These frameworks allow researchers to model how the behaviour of individuals within a social network can lead to collective outcomes that are not predictable from the behaviour of any individual alone. Through relational event simulations, researchers can study the effects of different network structures, parameters, and mechanisms on the emergence of social phenomena.

*Evaluate boundary conditions of theories* Evaluating the boundary conditions, or scenarios under which a theory makes sense is critical to the development and advancement of theories. Unfortunately, little is known about the boundary conditions of most social science theories. Knowing the boundary conditions of a specific theory can help guide the setting up of proper empirical experiments or the appropriate use of the theory in an empirical study. In our context, boundary conditions could refer to ranges of the parameter space of network effects, constraints on values of statistics, size of the network, or exogenous conditions that need to be present for a theory to be applicable. Also, knowing that a particular theory can only make valid predictions into the short future or only under fairly stable conditions informs the researcher how far into the future one can predict based on a fitted model or how much history must be taken into account in predicting future relational events in accordance with a specific theory. Simulations can be a powerful tool to explore the applicability of theories under various such conditions.

The approach here is to take a specific theory about social interaction that one intends to use in a research project and translate that into a relational event model. By simulating network interaction patterns across a range of parameter values or initial conditions, it can be assessed beyond which (combinations of) values the model starts to generate non-sensical or unrealistic dynamics such as the emergence of strongly separated subgroups, unrealistically high density, hyperactivity of actors with certain traits, odd speeding up and slowing down of interaction rates, etc. When appropriate characteristics of the simulated relational event network no longer pass the relevant sanity checks (either by being obviously nonsensical or by being quite removed from the dynamics of empirical reference data), it becomes clear that the theory that is mimicked by the relational event simulation no longer is realistic beyond these (combinations of) parameter values or initial conditions. This, then, provides the boundary within which a researcher would like to stay when using the theory to explain a phenomenon of interest.

*Incorporate timing and dynamism* The proposed simulation frameworks allow researchers to incorporate dynamism when developing theories by including feedback loops that are realized through endogenous statistics, time-varying exogenous covariates when attributes of actors and dyads can vary with time, time-varying parameters that can simulate realistic change-points, and memory when the influence of past events on new events decays with time. One advantage of this is that a theorist can assess what happens when a static social theory (which almost all established social theories to date are) is reformulated in an explicitly dynamic, time-sensitive manner. Although many of our current social theories are built on inherently dynamic ideas and arguments, they are rarely formulated (and validated) as such. It is often not at all obvious how to turn established static social theories into time-sensitive theories, since we have very little knowledge of how fast interactions develop, how long it takes until routine sets in, or how long emergent properties last. Simulating several dynamic versions of current social theories can provide hints to a theorist as to how an explicitly time-sensitive and dynamic version of the theory could be developed and put to the test.

### Group formation using optimal distinctiveness theory

As an example, we present a simulation approach to represent the Optimal Distinctiveness Theory using relational event simulations. The Optimal Distinctiveness Theory of [11] dictates that individuals have two fundamental and competing needs: (1) the need for inclusion, i.e. a desire to assimilate and interact with other individuals who share their social identity and (2) the need for distinctiveness from others in the actor’s surroundings. Individuals prefer to be identified with social groups that are neither too inclusive nor too distinctive, but are of optimal distinctiveness. These competing psychological mechanisms that operate at the actor level motivate the emergence of social groups that satisfy both needs at the same time [36]. Drawing upon optimal distinctiveness theory, we design an actor-based simulation experiment to observe the emergence of groups and their dynamics based on actor’s competing needs for optimal distinctiveness. We use an actor-oriented simulation approach, keeping in mind that the actor retains the agency to decide if and when to send events to other individuals in the network in accordance with the theory. Our simulation model assumes that actors make decisions about when to send an event in an attempt to satisfy their preference for interactions that are optimally distinct. Each actor *i* has an attribute $z_i$ (that reflects their fixed identity) and a preference for the optimal value of distinctiveness associated with that actor $d^{*}_i$. This value reflects the desired proportion of actors with whom *i* interacts and who do not share the same identity as *i*.

The distinctiveness of an actor $d_i$ at any time *t* is quantified with a proportion of distinct events, i.e. the total number of incoming and outgoing events that the actor *i* sends and receives from other actors who have an attribute value different from *i*’s divided by the total number of events sent and received by *i* from all other actors irrespective of the attribute:

7 $$\begin{aligned} d_i(t) = \dfrac{\sum \nolimits _{j \in \mathcal {A}/\{i\} } ( a_{ij}(t)+a_{ji}(t) ) \ \mathbb {I}[z_i \ne z_j ] }{\sum \nolimits _{j \in \mathcal {A}/\{i\} } ( a_{ij}(t)+a_{ji}(t) )}, \end{aligned}$$

where $a_{ij}(t)$ is the corresponding element of an $n \times n$ adjacency matrix *A*(*t*) containing the total number of past events between actors at time *t*. Recall from Sect. 2.2 that the DyNAM model requires two separate statistics for the sender and receiver choice, respectively. The distinctiveness aspect of an actor’s interaction preferences is incorporated into the simulation framework as a combination of a sender dissatisfaction statistic $X^s$ and a receiver choice statistic $X^r$.

Specifically, we operationalize the sender *i*’ dissatisfaction statistic $X^s(i,\mathcal {E}_t)$ as the absolute difference between the current distinctiveness and the optimal value of distinctiveness the actor seeks $| d_i (t) - d^*_i |$. A positive effect of the distinctiveness statistics implies that actors who are most dissatisfied with their current level of distinctiveness are most likely to be activated as the next sender in an attempt to adjust their distinctiveness level. We assume that for any actor, the direction of deviation from their optimal distinctiveness, i.e. towards more or less distinctiveness, does not influence the absolute level of dissatisfaction. Moreover, dissatisfied actors with a distinctiveness lower than their optimal level ($d_i (t) < d^*_i$) are likely to reach out to actors with attribute levels distinct from their own. Similarly, dissatisfied actors with a distinctiveness higher than their optimal level ($d_i (t) > d^*_i$) are likely to reach out to actors with the same attribute level as their own. If all actors are equally satisfied with their current distinctiveness level, an equilibrium will be maintained where all actors are equally likely to be sampled as the next sender [36].

For the DyNAM receiver choice model, a receiver choice statistic can be defined as follows.

8 $$\begin{aligned} X^r (j | i, \mathcal {E}_{t_m}) = {\left\{ \begin{array}{ll} \mathbb {I}[z_i = z_j] & \ d_i (t) > d^*_i \\ \mathbb {I}[z_i \ne z_j] & d_i (t) < d^*_i \end{array}\right. } \end{aligned}$$

In case $d_i (t) > d^*_i$, that is, when sender *i*”s interactions are more distinct than desired, the receiver statistic assigns a value of 1 for potential receivers with attribute the same as that of actor *i*. Similarly in case $d_i (t) < d^*_i$, i.e., when actor *i*’s interactions are more assimilated than desired, the receiver statistic assigns a value of 1 to receivers with attributes distinct from *i*’s. Otherwise, the statistics is set to 0. We further include an interaction of the above-defined statistic with an inertia receiver choice statistic in our model to ensure that receivers who share a greater volume of past events with a sender are more likely to be selected by the sender.

**Fig. 3:**
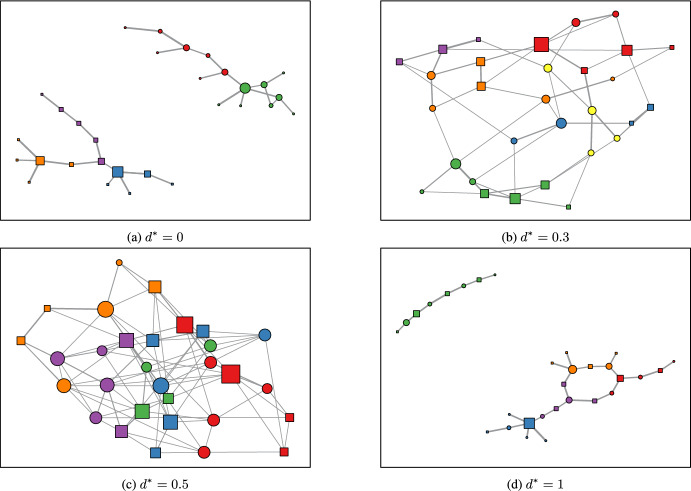
Simulated network plots for different optimal distinctiveness $d^*$ values. Square nodes represent nodes with $z=1$ and circular nodes represent nodes with attribute value $z=2$. The colours indicate the emergent groups to which the nodes were assigned based on the community detection algorithm. The size of nodes corresponds to the degree of the actors, and the width of the edges corresponds to the total volume of events exchanged in either direction between the two nodes

In our simulations, we assume that all actors have the same optimal distinctiveness value $d^*$. We simulate networks consisting of 30 actors with a binary attribute that represents their fixed identity $z=\{1,0\}$ for varying values of $d^*$. The proportion of actors with each attribute is equal. To identify emergent groups formed as a result of the simulations, we use the Louvain community detection algorithm [7]. This method of community detection essentially partitions nodes based on a greedy modularity optimisation. The groups identified from this method are considered emergent because they arise from interactions and decisions made by actors on the actor level, which lead to emergent group formation on a network level. Figure 3 shows the network plots for simulated sequences with different $d^*$ values. When $d^* = 0$, as illustrated in Fig. 3a, the actors in the simulation strongly prefer homophilic interactions, resulting in groups containing nodes of one attribute type. Actors with opposing attributes tend to cluster together rather than interact with actors of a different attribute.

For $d^* = 0.3$, shown in Fig. 3b, a mixed grouping is observed, with some larger groups containing both attribute types and some groups being homogeneous. The actors of opposing attributes interact with each other more often and the distance between them is shorter compared to the plot for $d^* = 0$. Figure 3 c for $d^* = 0.5$ shows a mixed grouping with an equal proportion of squares and circles in the groups, as well as a roughly random communication structure. The actors form a highly connected network, and actors with opposing attributes are roughly as close to each other as actors with the same attribute. In Fig. 3d where $d^* = 1$, there is a strong preference for distinctiveness among actors in their interactions, leading to a plot with mixed groupings. However, it is worth noting that the interactions within each group are bipartite, meaning that squares are only strongly associated with circles and vice versa.

These network plots pass our sanity checks, indicating that the specific way we translated the optimal distinctiveness theory into the relational event model is sound and does not produce unexpected results. We can further test the boundary conditions of the theory through simulations. In previous simulations, attributes were equally distributed between actors; however, this may not often be realistic. There may be an imbalance in the proportion of actors, for instance, when dealing with integration of minority communities or underrepresented sections of society at a university. To understand the effects of proportions of actors with different attributes on the network under the optimal distinctiveness theory, we simulated networks with 50 actors and varied the proportion of the minority within the population. The simulations were repeated 50 times for each combination of the proportion of minority actors $p \in \{0.1,0.2,0.3,0.4,0.5\}$ and for each value $d^*$ in the interval [0, 1].

**Fig. 4:**
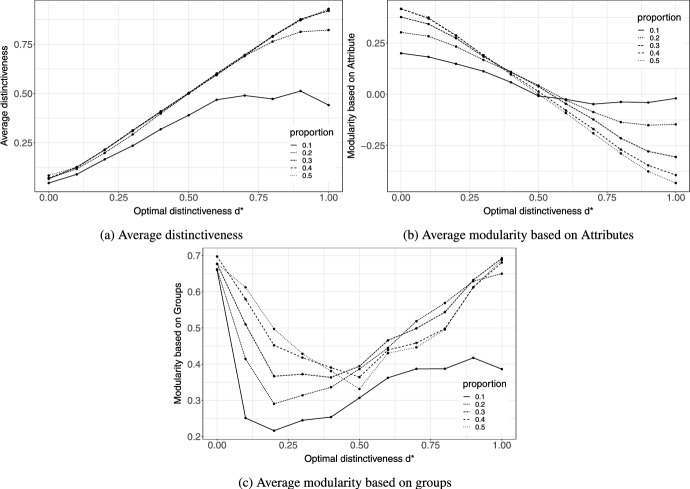
**a** Average distinctiveness of actors, **b** average modularity computed based on the partitioning of nodes by actor attribute and, **c** average modularity computed based on the partitioning of nodes by the detected groups at the end of 50 simulation runs for each value of optimal distinctiveness $d^* \in [0,1]$ and for each proportion of minority attribute

Figure 4a reports the average distinctiveness at the end of the simulations for the simulated event histories for the corresponding values *p* and $d*$. The results indicate that the average distinctiveness of the actors at the end of the simulations could converge to the optimal value when the proportions of the minority are greater than $10\%$. However, when $p=0.1$ the actors do not achieve their desired distinctiveness. When a minority is too small in size in relation to the population, actors are unsatisfied with their need for distinctiveness, particularly when $d^*$ is high.

Figure 4b presents the modularity values [41] computed based on the partitioning of the nodes by actor attribute at the end of the simulations. The value of the modularity reflects a measure of the strength of the partitions based on actor attributes, i.e., when modularity is high actors with the same attributes tend to cluster together and have a denser communication structure. The results show that with increasing $d^*$, the modularity values decrease. Indicating that at $d^*=0$ i.e. the actors in the simulation preferred only homophilic interactions, the simulated network indeed has high modularity. Whereas at $d^*=1$, when the preference for distinct interactions is at its peak, the modularity value is at its lowest. The decrease in modularity from $d^*=0$ to $d^*=1$ is less severe for lower proportions of minorities.

Figure 4c on the other hand, presents modularity values computed based on node partitioning, based on the groups detected by the community detection algorithm. When $d^*=0.5$ the modularity is at its lowest because the actors do not have a preference for distinctiveness. However, in each extreme, i.e. $d^*=$ 1 or 0 the clustering is high because actors have a strong preference to interact with actors with specific attributes, leading to tighter group formation. The modularity values for proportion $=0.5$ are fairly symmetric around $d^*=0.5$ however, for a lower proportion, the symmetry is altered with the minimum shifted to lower $d^*$ values. A possible explanation of this is that when $d^*$ is higher and the proportion of minority actors present in the network is lower, the clustering within a group is not strong due to the majority actors fulfilling their desired high distinctiveness from minority actors outside the group. The minimum at lower $d^*$ and lower *p* values occurs when the groups are formed around minority actors, thus the majority actors are able to fulfill their distinctiveness criteria within their groups.

Our purpose was to illustrate through a simple example *how* an interested researcher could articulate theories using the REM simulation framework. The results indicate how individual preferences of actors, in this case their desired distinctiveness, led to emergent group formation on the network level. We further tested a simple boundary for the theory by varying the proportion of minorities in the network. We see that dissatisfaction across actors is greater when the size of the minority group is skewed toward a very small minority presence compared to societies with larger minority groups, where actors are able to achieve their desired optimal distinctiveness. Future work remains to be done to investigate the impact of including more than two attributes or when varying the optimal distinctiveness in the population where certain actors have a higher desire for distinctiveness than others. Further, we assumed that the direction of dissatisfaction does not influence who is likely to be the next sender. We did not distinguish between actors who were unhappy with their distinctiveness and actors who were unhappy with their assimilation, as long as the absolute value of the dissatisfaction was equal. In reality, it is possible that these two cases may have a different impact on the probability of the next sender. Future work is needed to evaluate this. In addition, we also assumed that all actors had free choice and no restrictions in their communications; however, in reality, spatial or cultural restrictions could influence the interactions of actors in different ways and needs to be taken into account.

## Planning network interventions via simulations

Network interventions describe “the process of using social network data to accelerate behavior change or improve organizational performance” [51]. Interventions involve the use of social network data to bring about a behaviour change or to influence social dynamics in a network towards desirable outcomes. Previous work in network interventions focuses on the adoption of behaviour and the transmission of behavior between neighbors of nodes in a network, often using diffusion models [6, 50–52]. These models assume that behaviour is propagated in a network and once a node adopts a behaviour, it can influence it’s neighbour to adopt that behaviour. The edges between actors in such a network can represent the dissemination of information, opinions, ideas, goods, or even diseases. The variables of interest in diffusion models are often the proportion of nodes that have adopted a desirable behaviour or have acquired certain knowledge. However, in relational event models, the variable of interest is the rate of interaction that describes who interacts with whom and when. REM or DyNAM can be utilized to model interventions in evolving interaction networks where the intervention aims to influence the interaction dynamics itself.

The relational event simulation framework can be used to predict or better understand the possible impact of intervention outcomes over time. This can be of great practical use, for example, by allowing managers to try out several interventions “in-silico” before deciding which one(s) to try out “in-practice”; the effects of competing interventions can be assessed without spending much money or other resources and without overhauling an organization based on a simple managerial hunch. Rather, a manager can get a fairly good idea of which intervention is likely to work, how it should be implemented, and what the pitfalls are that need to be monitored when the intervention is implemented in real life.

Ideally, the simulation is initialised from an estimated model based on an empirical observed network. This allows a realistic simulation of the intervention based on empirically derived conditions and effect estimates. Adams and Schaefer [1] explored how the same intervention can have different results depending on the initial conditions of the network. This is also applicable in the REM context. For example, consider an intervention that is conducted for a while and aims to promote interdepartmental collaboration in an organizational network. If the network is characterized by high tendencies towards inertia, the interdepartment interactions that were originally triggered by the intervention (that only ran for a limited time) may become sustained into the long term due to the network’s tendency for inertia. However, if inertia is only low (or negative) in this network, it is possible that, once the intervention ends, the network reverts back to its original state. Having an estimate of the effects operational in the empirical network beforehand would be highly beneficial in planning interventions. The simulations could then be carried out to evaluate the outcomes of planned interventions that explore the implications of a manipulated set of effects or initial conditions.

### Types of network interventions

In order to effectively simulate realistic interventions using the simulation framework, it is important to understand the types of interventions that can be simulated in the context of relational events and how the mathematical modification of the model translates to its implementation in practice. To be able to effectively translate an intervention into the REM or DyNAM model, it is important to define which aspects of the model the intervention modifies. To facilitate this, we distinguish between three types of REM interventions: i) Actor and edge attributes, ii) Network Effects, and iii) Structure - based on the locus of change in the model.

- Attributes: Interventions may involve modifications to attributes of actors or relationships between actors (dyadic attributes) to answer questions such as: Can changing the layout of desks lead to a change in the communication patterns of students in a classroom? What is the effect of changing the gender or hierarchy distribution on communication in an organization? The analytical translations of such interventions are straightforward because they involve simply modifying the actor or dyadic attributes while keeping other aspects of the model the same as preintervention.
- Network effects: Interventions of this type focus on modifying network effects of the model itself by either changing the magnitude of network effects (by adjusting the corresponding parameters) or by removing (or adding) network effects that may (or may not) be operative in the social network previously. For example, consider an intervention that is carried out by organising social events designed to increase mixed-gender contact opportunities among university students. The analytical translation of this intervention could be a temporary increase in the magnitude of the effect of gender heterophily. Simulating events beyond the duration of the intervention could reveal the long-term effects of the designed intervention on the ties between students. Another example of this type of intervention is the reduction in the popularity of a smoker, as students in a university are made aware of the harmful effects of smoking. The translation of this effect could be a decrease in magnitude of the effect for the smoker popularity statistic (interaction of the smoker attribute and the in-degree of the receiver).
- Structure: Interventions that change the structure of a social network involve changes in the size of the network by the addition or removal of actors, the risk set by restricting which pairs of actors may interact, and changes in the composition and grouping of actors. Examples of structural changes include the addition of employees when companies merge or acquire one another, the removal of a key leader in an organization, and the reassignment of teams and managers when new projects are started. The analytical translation of these interventions is straightforward and often involves making alterations to the risk set. In case of re-assignment of teams for instance, dyads with employees from different teams or locations may no longer be considered in the riskset.

The three categories above are not disjoint, as interventions in practise can involve multiple categories. For example, consider an intervention that involves the addition of sport activities in a university. Students and staff from different departments would now have the opportunity to interact with each other during these events. The analytical translation of such an intervention involves all the three categorizations defined above. Actor attributes that represent whether or not individual actors participate in sports and the type of sport if they do, are added to the model. A new network effect can be added for homophily interactions that may take place between actors attending the same sports sessions. In addition, the structure of the risk set can also be altered to include dyads from different departments that participate in the same sports that may not previously be considered at risk of interaction.

**Fig. 5:**
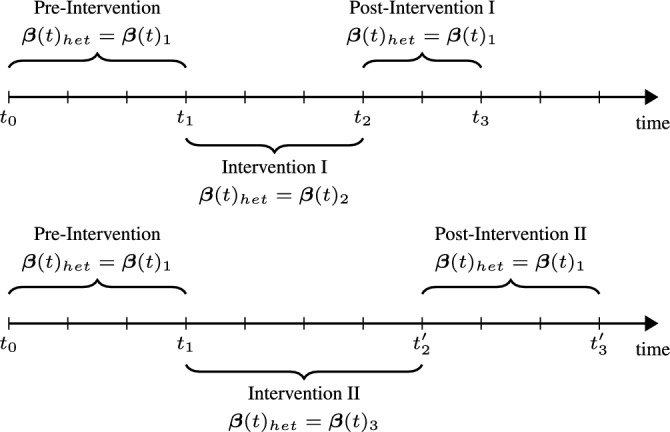
Diagrammatic representation of two interventions with varied intervention duration and strengths. In Intervention I, $\varvec{\beta }(t)_{het}$ increases from $\varvec{\beta }(t)_1$ to $\varvec{\beta }(t)_2$ during the interval $(t_1, t_2)$ and reverts to $\varvec{\beta }(t)_1$ at $t_2$. In Intervention II, the effect is increased to $\varvec{\beta }(t)_3$ over a longer period $(t_1, t_2')$, before returning to $\varvec{\beta }(t)_1$ at $t_2'$

### Evaluating the persistence of intervention outcomes

In this example, our aim is to explore the longevity of the outcomes of different interventions through simulations. With simulations, it is possible to assess how a network would react to competing interventions and helps in improving intended network interventions towards a desirable outcome. The intervention in this example is intended to increase the interdepartment collaboration between employees of two departments in an organization. The number of interdepartmental emails is used as a proxy for the collaboration across departments. This intervention example is a simplified version of an actual intervention study that we conducted for the R&D department of a large consumer goods company in western Europe. To increase the interdepartment communication, the organization intended to intervene by providing the employees with incentives to increase their inter-department communication (e.g., by organizing new multidepartment projects and organising joint meetings). This intervention is modelled in our example by temporarily increasing the departmental heterophily effect $\varvec{\beta }(t)_{het}$ in the model. Our interest is to evaluate what the effect is of the intervention after it ends and how long the increased heterophily effect remains in the network after the intervention has ended and the network has returned to its “normal” dynamic. In this analysis, we assume that the intervention does not structurally change the norms or preferences of the employee interaction. This allows us to focus on the immediate and direct effects of the intervention on event dynamics while keeping broader behavioral patterns constant. Therefore, we apply the preintervention model to postintervention interactions (although the histories will have been altered by the intervention itself). Other assumptions, such as long-term changes in interaction preferences, could also be explored in other simulations to assess alternative counterfactual scenarios.

Before testing the interventions using simulations, we first estimate the coefficients of a model based on interactions before the intervention, for a period spanning 243 days using a standard REM. The REM model contains various endogenous effects (represented by $\varvec{\beta }(t)_{endo}$) such as inertia, reciprocity, participation shifts, and degree effects. Exogenous effects (represented by $\varvec{\beta }(t)_{exo}$) for the gender, seniority and subdomain are also included. An additional effect for inter-departmental heterophily ($\varvec{\beta }(t)_{het}$ is also included and is the locus of the intervention.

The model fits the data reasonably well, and we assume that it captures the way in which the company’s employees tend to interact well. In the following, we will compare nine versions of the intervention to increase interdepartmental homophily (by increasing $\varvec{\beta }(t)_{het}$). The interventions differ in the length of time they run and in the strength of the intervention, and we aim to evaluate the persistence of the intervention effects after the intervention has been carried out. Figure 5 graphically shows two examples. Intervention I lasts from $t_1$ to $t_2$ and for this period we increase $\varvec{\beta }(t)_{het}$ (the parameter for the heterophily effect) to $\varvec{\beta }(t)_2$. Alternatively, intervention II runs longer than intervention I (from $t_1$ to $t_2'$) and has a different heterophily parameter $\varvec{\beta }(t)_{het}=\varvec{\beta }(t)_3$.

**Fig. 6:**
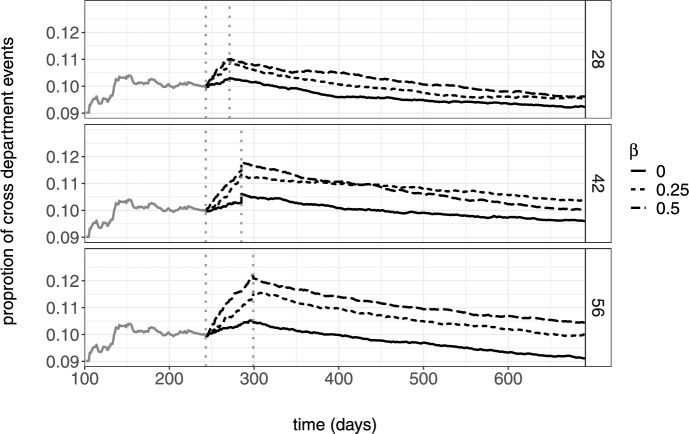
Median proportion of inter-departmental events simulated after an artificial intervention. The vertical dotted lines indicate the intervention period. Top panel shows the simulations for an intervention lasting 28 days. The middle panel with a duration of 42 days and the bottom panel with 56 days. The parameter $\beta$ refers to the heterophily effect $\varvec{\beta }_{het}$, which was varied during the intervention

Using the pre-intervention sequence as the initialization, we simulate the relational events in the network for the length of time the intervention runs, and following the intervention, we further simulate the relational sequence for 50 weeks after each intervention. We consider three durations of the intervention $\{28,42,56\}$ days and three sizes of inter-departmental heterophily effect $\{0,0.25,0.5\}$. Therefore, we generate relational event sequences under $3 \times 3 = 9$ different interventions. Since the simulations have a stochastic component, we generate 50 relational event sequences for each intervention and compute the proportion of interdepartmental events in each. For each intervention, we report the median proportion in the 50 simulated sequences in Fig. 6. The proportion of interdepartment events increased the most for interventions held for the longest period and for the strongest effect size. The effects of the interventions on the inter-departmental communication lasted 38 days for the case of $\varvec{\beta }(t)_{het}=0$, 91 days for the case of $\varvec{\beta }(t)_{het}=0.25$ and 208 days when $\varvec{\beta }(t)_{het}=0.5$ when the duration of the intervention period was 28 days. Similarly, the effects of the intervention last 61 days for $\varvec{\beta }(t)_{het}=0$, 247 days for $\varvec{\beta }(t)_{het}=0.25$, and 283 days for $\varvec{\beta }(t)_{het}=0.5$ when the duration of the intervention was 42 days. Furthermore, the impact of the intervention lasted 72 days for $\varvec{\beta }(t)_{het}=0$, 307 days for $\varvec{\beta }(t)_{het}=0.25$, and persist beyond our simulation period when $\varvec{\beta }(t)_{het}=0.5$ when the duration of the intervention was 56 days. We conclude that relational event simulations can provide a useful tool in planning and designing network interventions, as well as to evaluate the persistence of interventions outcomes over time.

### Evaluating intervention targeting strategies

Our second example concerns the situation in which one would like to know at whom to aim a specific intervention. For example, in organisational networks, it may be prohibitively costly and complicated to intervene directly in everyone’s work. Rather, it may often be effective to target an intervention at a limited set of employees only, acknowledging that their changed behaviour will subsequently affect that of their coworkers, causing the intervention effect to spread across the network naturally. Of course, the effectiveness of this will usually depend on the set of employees to which the intervention is aimed. This is what we will show an example of here. Again, the intervention is intended to increase interdepartmental collaboration.

**Table 2:** Overview of measures used to select intervention targets

Targets	Description
1. Random	Each actor has equal chance of selection
2. Highest centrality	Actors with the highest betweenness centrality are selected
3. Lowest centrality	Actors with the lowest betweenness centrality are selected
4. Highest inter-department out-degree	Actors with the highest number of inter-departmental out-going events are selected
5. Lowest inter-department out-degree	Actors with the lowest number of inter-departmental out-going events are selected
6. Senior	Actors designated as ‘Senior’ in the dataset (based on their tenure at the organization) are selected
7. Junior	Actors designated as ‘Junior’ in the dataset (based on their tenure at the organization) are selected
8. Male	Actors designated as ‘Male’ in the dataset are selected
9. Female	Actors designated as ‘Female’ in the dataset are selected
10. No intervention	No intervention was carried out

Keeping the duration of the intervention constant, we consider different targets for the intervention. Table 2 provides an overview of the nine different sets of targets that we consider. The “random targets” intervention is used as a baseline to compare the other, more specifically targeted, interventions against. Interventions 2–5 use the endogenous activity of actors as a criteria for target selection. Interventions 6–9 are based on exogenous attributes of the actors. We also include a scenario where no intervention is carried out.

The interventions in our example are carried out by modifying the department-heterophily parameter $\varvec{\beta }(t)_{het}$ only for the selected targets. Specifically, the departmental heterophily parameter during the intervention remains the same as its value prior to the intervention ($\varvec{\beta }(t)_{het}^{pre} = -0.58$) for dyads where the sender does not belong to the target group of actors $\mathcal {Q}$. However, the heterophily parameter is increased to a higher value ($\varvec{\beta }(t)_{het}^{intv} = 0.5$) for dyads where the sender belongs to the target group of actors. Thus, the heterophily effect is split into two groups with two different parameter values.

$$\begin{aligned} \varvec{\beta }(t)_{het}(i,j) = {\left\{ \begin{array}{ll} \varvec{\beta }(t)_{het}^{pre} & \ i \notin \mathcal {Q}^{K} \\ \varvec{\beta }(t)_{het}^{intv} & \ i \in \mathcal {Q}^{K} \end{array}\right. } \end{aligned}$$

**Fig. 7:**
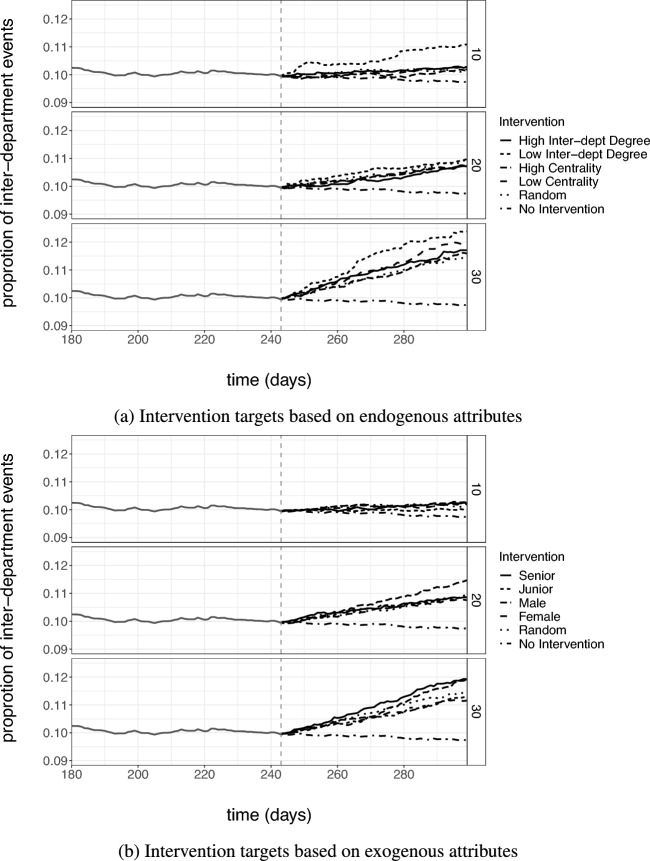
Median proportion of inter-departmental events simulated for all the intervention strategies. **a** Intervention targets are selected on endogenous attributes, and **b** targets are selected on exogenous attribute. The panel in each sub-figure shows the results from top to bottom, K = {10%, 20%, and 30%}

In addition to comparing the different strategies for target selection, we also vary the fraction of actors (*K*) that are selected for each strategy (from the set of 60 employees). Figure 7a shows the reported median proportion of simulated interdepartment events for endogenous selection strategies, random selection, and no intervention for K = 10%, 20%, and 30%. For $K=10\%$ and $20\%$ actors selected based on having the lowest degree between departments had the greatest impact on the proportion of interdepartmental communication, while selecting targets based on the remaining strategies impacted the proportion of interdepartmental somewhat equally as selecting random targets. When $K = 30 \%$, the low inter-departmental degree strategy is again leading followed by the high centrality. The remaining targeting strategies impacted the proportion of inter-departmental events somewhat equally. Figure 7a depicts the comparison among exogenous attribute-based strategies. The targeting of seniors or women has the greatest impact for $K=30\%$, while the strategies were roughly tied when fewer actors were selected, that is, $K=10\%$. In all cases, targeting junior employees had the least effect. This may be due to juniors having fewer established connections or less influence over broader communication patterns, making their behaviour less likely to ripple through the network.

The simulation results indicate that targeting a smaller set of actors rather than the entire network can indeed lead to desirable results in that network. However, the selection of the intervention targets can have a considerable impact on the results. In our example, we found that targeting juniors does not produce compelling results, rather seniors in the organization should be targeted for promoting interdepartmental communication. Further targeting actors who have a lower interdepartmental communication rate can prove to be more impactful than actors with higher interdepartmental communication or even higher centrality. This may be because the marginal gains from activating previously inactive or weakly connected actors can be greater than intervening on already active ties. Hence, when designing an intervention in practise, relational event simulations can be an effective tool for tailoring intervention design to maximize network-level change.

## Evaluate model sensitivity via simulations

Simulations based analysis can be used to assess the sensitivity of relational event models to varying model specification and assumptions. By generating data under controlled conditions, researchers can systematically examine how well a model recovers the underlying data-generating structure or how deviations from the true data-generating process affect inference. This can be particularly useful to testing and benchmarking novel REM extensions. Thus, by utilising simulations, researchers can clarify under what conditions new extensions of REM remain reliable.

In addition to sensitivity analysis, simulations also play a crucial role in power analysis, allowing researchers to evaluate the certainty of detecting true effects under various conditions. Unlike analytical approaches that rely on theoretical assumptions, simulation-based power analysis accounts for complex dependencies in data such as time-varying effects, memory effects, or interaction terms for which it may be difficult to assess their effect on power analytically. By varying various aspects such as effect sizes, number of actors, scaling, or other model specifications, researchers can determine the conditions under which a model is likely to produce reliable estimates (cf., [44]).

**Fig. 8:**
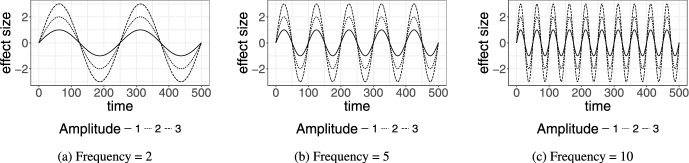
Time-varying inertia effect modelled as a sinusoidal function with varying amplitudes and frequency. Panels **a**–**c** correspond to increasing frequencies of 2, 5, and 10, respectively, while the amplitude increases from 1 to 3 within each panel

### Evaluate sensitivity of a time-varying effect model

Due to the complexity of social interaction behaviour in real-life networks, it is very likely that drivers of social interaction behaviour, such as inertia or reciprocity, change over time [e.g., 40]. To assess the sensitivity of relational event models (REM) to the misspecification of time-varying effects, we conduct a series of simulations in which we generate relational event data with a time-varying inertia effect and then fit a standard REM that assumes a constant inertia effect. This will allow us to evaluate how well the misspecified model captures the true underlying model and the extent to which bias is introduced in other model parameters.

The simulations involve generating relational event sequences from a tie-oriented REM, where the inertia effect varies over time according to sinusoidal patterns that vary in frequency and amplitude according to Equation 9. We simulate data for a network of $N = 10$ actors up to time 500. For each combination of frequency $f \in \{2,5,10\}$ and amplitude $a \in \{1,2,3\}$, a time-varying inertia function is created and 50 independent runs are performed for each combination of amplitude and frequency.

9 $$\begin{aligned} \beta _{inertia}(t) = a \cdot \sin \left( 2\pi \frac{f}{T} t \right) \end{aligned}$$

where *f* is the frequency, *a* is the amplitude and *T* is the maximum time for simulations (set to $T=500$). The time-varying effect function can be visualized in Fig. 8. For the remaining effects in the model, we used constant parameters. We include reciprocity, activity (i.e. out-degree of the sender), popularity (i.e in-degree of the receiver), and two participation shift statistics: psABBA and psABXA. The corresponding effects are : $\beta _{\text {baseline}} = -3$, $\beta _{\text {reciprocity}} = 0.6$, $\beta _{\text {outdegreeSender}} = 0.5$, $\beta _{\text {indegreeReceiver}} = 0.2$, $\beta _{\text {psABBA}} = 0.6$, $\beta _{\text {psABXA}} = 0.4$ .

We explore the impact of this misspecification by evaluating the recall, which reflects how well the model would fit the generated data, and bias in the estimates of other constant effects. To compute the recall, we calculate the estimated rate for each dyad according to the fitted model and rank the dyads in decreasing order of event rate. We assign rank 1 to the dyad with the highest estimated rate, rank 2 to the dyad with the second largest estimated rate, and so on. At each time point, we observe the rank of the dyad that actually occurred in the observed event sequence. If the rank of the observed event is greater than a threshold percentile, that event is considered correctly predicted by the model. The proportion of correctly predicted events (also known as * recall*) can be used as a measure of predictive performance [18, 24, 33] to evaluate the goodness of fit of relational event models.

**Fig. 9:**
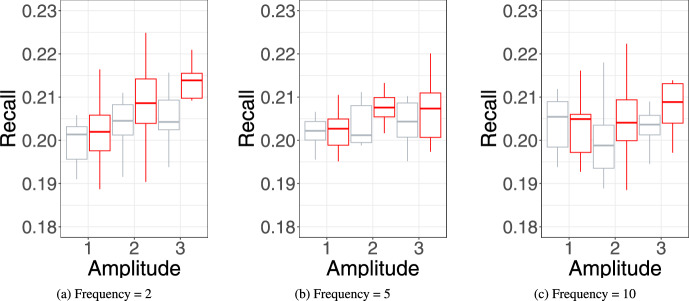
Recall (i.e. the proportion of correctly predicted realized events by the model) across different frequencies and amplitudes (based on 50 simulated event sequences per frequency and amplitude combination). The time-varying inertia model is in red and the constant-inertia model in grey

Figure 9 illustrates the recall with the 95th percentile as the threshold under two conditions: using the data generating a time-varying inertia model (shown in red) and a misspecified constant-inertia model fitted on the simulated event sequences (shown in gray), across different frequencies and amplitudes of the inertia effect. Across the three frequency conditions (2, 5, and 10), the time-varying model consistently achieves higher recall than the constant-effect model, indicating that correctly specifying the inertia effect improves fit. As amplitude increases, recall tends to increase for both models, but the gap between the time-varying and constant-effect models remains due to the impact of misspecification being more pronounced when the inertia effect exhibits stronger fluctuations. Interestingly, amplitude appears to have less influence on recall when the frequency is high. This may be explained by the fact that at very high frequencies (relative to the interevent time), rapid oscillations average out over time reducing the impact of the variation.

**Fig. 10:**
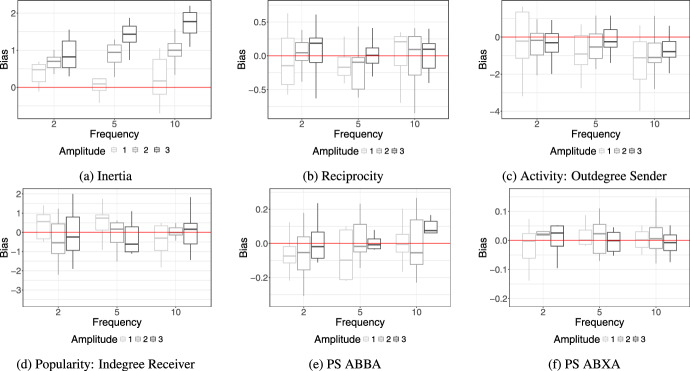
Bias for the constant-effect statistics when the time-varying inertia effect is misspecified as constant (based on 50 simulated event sequences per frequency and amplitude combination). Each panel reports the distribution of estimation bias for a different statistic: **a** inertia, **b** reciprocity, **c** activity (outdegree sender), **d** popularity (indegree receiver), **e** participation shift ABBA, and **f** participation shift ABXA. The red horizontal line indicates zero bias. Bias is shown across combinations of three amplitudes (1, 2, 3) (light grey to dark-grey) and three frequencies (2, 5, 10) of the underlying time-varying inertia

Furthermore, Fig. 10 illustrates the impact of the misspecification of inertia on the other endogenous statistics in the model. The results indicate that bias in the estimates is influenced by both frequency and amplitude, with some statistics being more affected than others. Reciprocity and popularity (indegree receiver) show larger variations, suggesting that their estimates are less stable across different frequencies and amplitudes. In contrast, the participation shifts ABBA and ABXA exhibit relatively smaller biases, indicating that these effects are estimated more reliably and are less prone to misspecification of the inertia statistic in this model. Furthermore, the bias in the Inertia effect itself can be seen in Fig. 10a. The bias in inertia is substantial for higher frequency and increases further with increasing amplitude. This is because higher frequency and amplitude lead to greater variability in the time-varying inertia effect, making it harder for the constant-effect model to capture the underlying pattern accurately and resulting in increasingly biased estimates.

The purpose of this illustration was to highlight how simulations can be utilized to perform sensitivity analysis in the event of misspecified REMs. Such analyses can provide researchers with insights into the robustness of their REMs when the assumptions, such as those about the temporal homogeneity of the network effects, i.e., that the effects are constant over time, are violated.

## Making predictions via simulations

Relational event models can be used not only for inference but also for prediction. By simulating from a fitted model, researchers can explore how event sequences can unfold beyond the observation window, predict the timing of future interactions, or evaluate possible outcomes under different conditions. This approach is particularly useful in applied settings, such as forecasting communication patterns, monitoring risk, or anticipating conflict scenarios.

However, it is important to note that relational event prediction is typically probabilistic rather than deterministic, especially when forecasting beyond the next few events. Individual simulated sequences may differ from reality, yet the model can still produce accurate forecasts on average. This reflects a well-known issue in time series forecasting: even if specific predictions are “wrong,” the model may still be well-calibrated across many draws [23, 25]. In this section, we illustrate how simulations from a fitted model can support predictive analysis using an empirical case involving policing and the dynamics of criminal gang violence.

### Predicting gang violence from a fitted model

Sect. 3.1 assessed the goodness-of-fit of the model presented by [24], we now turn to understanding what the model predicts about gang conflicts. Rather than checking how well the model explains the past, this section explores its substantive predictions: What dynamics emerges when the model is allowed to generate sequences of events beyond the observation period. Using the fitted relational event model developed by [24], we simulate 200 independent sequences of gang violence in Los Angeles that extend the relational event history in these data.

**Fig. 11:**
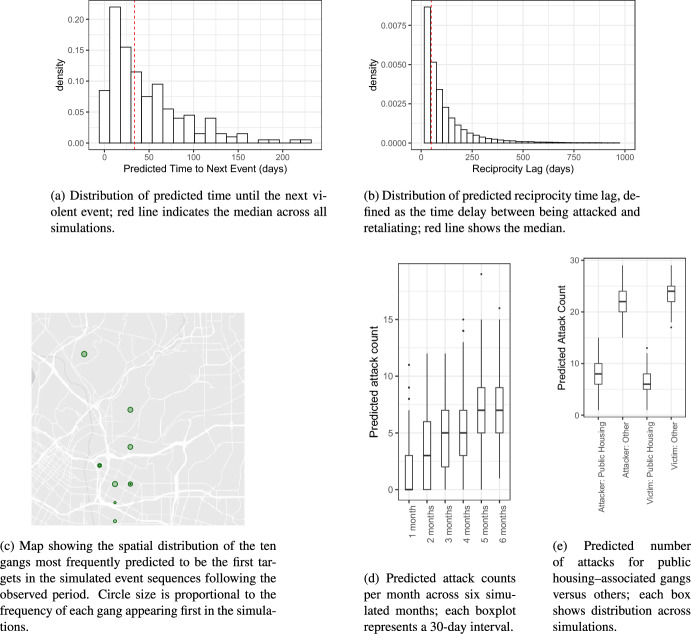
Simulated predictions from the relational event model of gang violence in Los Angeles. Each subplot visualizes a different predicted quantity based on 200 simulated event sequences generated from the fitted model of [24]

Figure 11 summarizes several structural and temporal characteristics derived from the simulations. The spatial distribution of the gangs that are most likely to be targeted in the next event following the observed period is shown in Fig. 11c. The map reflects the top ten gangs that appeared the most frequently as the first predicted target across all simulated event sequences. The distribution of predicted time to next event is shown in Fig. 11a. The median time to the next event across the simulations was 33.75 days. Reciprocity lag, defined as the delay between an attack by a gang and the subsequent transmission of an event, is plotted in Fig. 11b. The monthly predicted event counts are summarized in Fig. 11d.

Finally, Fig. 11e compares predicted event participation by gangs associated with public housing versus others. The median counts among gangs associated with public housing for both the sender and receiver roles are lower than the others.

Although relational event models provide a structured framework for modelling and simulating complex social interactions such as gang violence, their use as predictive tools for real-world decision-making remains an open area of investigation. More work is needed to evaluate the temporal stability and validity of the out-of-sample models. For policy and law enforcement contexts, REM-based predictions must be interpreted with caution and complemented by contextual background knowledge and understanding of the structural mechanisms that underlie patterns of violence.

## Discussion

Relational event simulation techniques are indispensable tools for social network researchers for numerous purposes such as making predictions, building theories, testing model assumptions, evaluating hypotheses on network dynamics, or checking statistical power. To apply these techniques in a relatively easy manner, the paper presented flexible simulation frameworks under dyadic and actor-oriented relational event models which are readily available using the R package remulate. The usefulness of simulation techniques using remulate was shown for different types of problems in social network research. First, the package was used for assessing the goodness of fit of relational event models to study violent interactions between criminal gangs. Simulations indicated that, while the model fits well in general, it may underestimate inter-event time between escalations in gang conflicts, suggesting a need for model refinement to better capture these dynamics. Second, the simulation framework was used to build, evaluate, and extend social network theories, one of the most important goals of social research. This was demonstrated through the application of the Optimal Distinctiveness Theory, illustrating how individual preferences for inclusion and distinctiveness drive group formation in networks. Third, the simulation framework was used to plan network interventions in real life, identify intervention points, and inform policy decisions. For example, simulations helped design interventions to increase interdepartment communication in an organization, evaluating the persistence and impact of these intervention outcomes over time. Fourth, simulations were used to test the sensitivity and robustness of relational event models to model misspecification. We simulated data from a model with a time-varying effect and compared estimation outcomes using both correctly specified time-varying effects and standard constant-effects models. Fifth, the framework was used to illustrate how relational event simulations can be applied to prediction. In a case study on gang violence, simulations were used to estimate when and between whom future violent events might occur, showing how fitted models can be used to generate plausible future event sequences beyond observed dynamics.

Although flexible frameworks and software provide a practical means for social network researchers to simulate realistic dynamic networks for different purposes, the application of the methodology remains challenging. First, the task of specifying appropriate simulation models is not trivial and time consuming. For this reason, the use of existing theories (even on a general level), contextual understanding, and extensive exploration is essential to avoid model misspecification. Simulations from a misspecified model may result in unrealistic networks such as networks with unrealistically high (or low) connectivity. Network simulation techniques are indispensable to avoid this problem. Second, the simulations we outlined in our applications provide only control over the local mechanisms of interaction. These local ’rules’ give rise to global network-level properties indirectly. However, our simulations do not give direct control over global network-level properties. For example, if a network of a specific density or degree distribution is desired, then the methods proposed in this paper may not be helpful, as the density or degree distribution of a relational event network only partially depends on the effects used to simulate relational event sequences. Despite these challenges, statistical simulation techniques still belong to the best and most flexible methods of a social network researcher toolkit to study social interaction dynamics in complex real-life applications.

Finally, there are various important directions of future research including a comprehensive discussion of the impact of statistics and their (highly) inherent multicollinearity when building relational event models, both for generating relational event data as well as for model fitting to empirical data. This important but challenging aspect includes a thorough assessment of the impact of the scaling method, the choice of the decay functions (to capture memory retention), and the interpretation of the size of REM coefficients given the dependency of the corresponding statistics. There have already been important contributions to these topics (e.g., Perry & Wolfe, 2009; DuBois et al. 2013, Arena et al., 2023, to name a few) on which can be built. For example, to avoid waiting times converging to zero in the long run (a form of degeneracy), scaled or normalized endogenous statistics, which are bounded, are recommended over statistics based on raw counts, which are unbounded.

Moreover, extending the simulators further to account for more complex heterogeneity structures in network data is recommended. Extensions include (crossed) random effects models or latent variable models [17, 39], more complex shapes of memory decay in endogenous statistics, and further refining time-sensitive goodness-of-fit indices. The simulation objectives and examples discussed in this article along with the easy-to-use open-source R package remulate can provide the reader with a tool that opens up new avenues of research and shortens the design time for simulating time-stamped relational event data.

## Figures and Tables

**Fig. 12:**
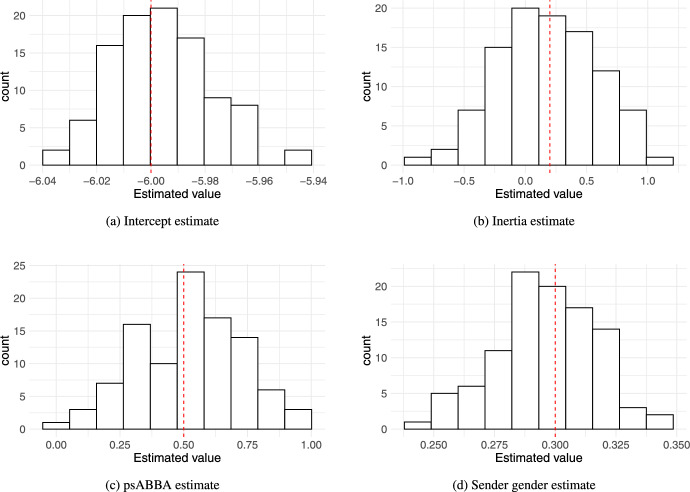
Sampling distributions of parameter estimates across 100 simulations. Dashed red lines indicate the true parameter values

## Data Availability

Data utilized in Sect. 4, 5.2, 5.3 and 7 are not publicly available. The simulation scripts are available upon request.

## Appendix 1: Validity of simulations

To evaluate the validity of the simulation procedure implemented in remulate, we conducted a controlled experiment in which we simulated 100 relational event sequences from a known tie-oriented model that included effects for baseline rate, inertia, participating shift psABBA, and a sender covariate. We then fit the same model specification to each simulated dataset using remstimate [3], and plotted the sampling distributions of the estimated parameters. The results show that the estimated values are tightly centred around the true parameters used in simulation, indicating that the simulation procedure produces event sequences that faithfully reflect the intended model (Fig. 12).

The R code related to the validity experiment is as follows:

## Footnotes

1 Without loss of generality, we keep the set $\mathcal {A}$ fixed over time in this paper.

2 Relational Event Network, Relational Event Histories, and Relational Event Sequence are referred to analogously.

3 Tests were conducted on a Dell Latitude 5400 laptop with an Intel Core i7-8665U (8 cores), 16GB RAM, running Ubuntu 22.04.4 LTS.

## References

[1] Adams, J., & Schaefer, D. R. (2016). How initial prevalence moderates network-based smoking change: Estimating contextual effects with stochastic actor based models. *Journal of Health and Social Behavior,**57*(1), 22–38. 26957133 10.1177/0022146515627848 PMC6679597

[2] Amati, V., Lomi, A., & Snijders, T. A. B. (2024). A goodness of fit framework for relational event models. *Journal of the Royal Statistical Society Series A: Statistics in Society,**187*(4), 967–988.

[3] Arena, G., Lakdawala, R., & Generoso Vieira, F. (2023a). Remstimate: Optimization frameworks for tie-oriented and actor-oriented relational event models. Retrieved from https://github.com/TilburgNetworkGroup/remstimate

[4] Arena, G., Mulder, J., & Leenders, R. T. A. (2023b). How fast do we forget our past social interactions? understanding memory retention with parametric decays in relational event models. *Network Science,**11*(2), 267–294.

[5] Arena, G., Mulder, J., & Leenders, R. T. A. (2024). A bayesian semi-parametric approach for modeling memory decay in dynamic social networks. *Sociological Methods & Research,**53*(3), 1201–1251.

[6] Badham, J., Kee, F., & Hunter, R. F. (2021). Network structure influence on simulated network interventions for behaviour change. *Social Networks,**64*, 55–62.

[7] Blondel, V. D., Guillaume, J.-L., Lambiotte, R., & Lefebvre, E. (2008). Fast unfolding of communities in large networks. *Journal of Statistical Mechanics: Theory and Experiment,**2008*(10), P10008.

[8] Box, G. E. P. (1976). Science and statistics. *Journal of the American Statistical Association,**71*(356), 791–799. 10.1080/01621459.1976.10480949

[9] Brandenberger, L. (2019). Predicting network events to assess goodness of fit of relational event models. *Political Analysis,**27*(4), 556–571.

[10] Brandes, U., Lerner, J., & Snijders, T. A. (2009). Networks evolving step by step: Statistical analysis of dyadic event data. In *2009 international conference on advances in social network analysis and mining* (pp. 200–205).

[11] Brewer, M. B. (1991). The social self: On being the same and different at the same time. *Personality and Social Psychology Bulletin,**17*(5), 475–482.

[12] Butts, C. T. (2008). A relational event framework for social action. *Sociological Methodology,**38*(1), 155–200.

[13] Butts, C. T. (2023). *relevent: Relational event models*. R package version 1.2-1.

[14] Butts, C. T., & Marcum, C. S. (2017). A relational event approach to modeling behavioral dynamics (pp. 51–92). arXiv:1707.09902 [stat]

[15] Corning, P. A. (2002). The re-emergence of “emergence’’: A venerable concept in search of a theory. *Complexity,**7*(6), 18–30.

[16] Davis, J. P., Eisenhardt, K. M., & Bingham, C. B. (2007). Developing theory through simulation methods. *Academy of Management Review,**32*(2), 480–499.

[17] DuBois, C., Butts, C., & Smyth, P. (2013a). Stochastic blockmodeling of relational event dynamics. In *Proceedings of the sixteenth international conference on artificial intelligence and statistics* (pp. 238–246). PMLR. ISSN: 1938-7228.

[18] DuBois, C., Butts, C. T., McFarland, D., & Smyth, P. (2013b). Hierarchical models for relational event sequences. *Journal of Mathematical Psychology,**57*(6), 297–309.

[19] Epstein, J. M., & Axtell, R. L. (1996). *Growing artificial societies: Social science from the bottom up*. The MIT Press.

[20] Falzon, L., Quintane, E., Dunn, J., & Robins, G. (2018). Embedding time in positions: Temporal measures of centrality for social network analysis. *Social Networks,**54*, 168–178.

[21] Gibson, D. R. (2003). Participation shifts: Order and differentiation in group conversation. *Social Forces,**81*(4), 1335–1380.

[22] Gilbert, G. N., & Troitzsch, K. G. (2005). *Simulation for the social scientist* (2nd ed.). Open University Press.

[23] Gneiting, T., Balabdaoui, F., & Raftery, A. E. (2007). Probabilistic forecasts, calibration and sharpness. *Journal of the Royal Statistical Society: Series B (Statistical Methodology),**69*(2), 243–268.

[24] Gravel, J., Valasik, M., Mulder, J., Leenders, R., Butts, C., Brantingham, P. J., & Tita, G. E. (2023). Rivalries, reputation, retaliation, and repetition: Testing plausible mechanisms for the contagion of violence between street gangs using relational event models. *Network Science,**11*(2), 324–350.

[25] Hamill, T. M. (2001). Interpretation of rank histograms for verifying ensemble forecasts. *Monthly Weather Review,**129*(3), 550–560.

[26] Hanneke, S., Fu, W., & Xing, E. P. (2010). Discrete temporal models of social networks. *Electronic Journal of Statistics,**4*(none), 585–605.

[27] Holland, P. W., & Leinhardt, S. (1977). A dynamic model for social networks. *The Journal of Mathematical Sociology,**5*(1), 5–20.

[28] Hunter, D. R., Goodreau, S. M., & Handcock, M. S. (2008). Goodness of fit of social network model. *Journal of the American Statistical Association,**103*(481), 248–258.

[29] Juozaitienė, R., & Wit, E. C. (2024). Nodal heterogeneity can induce ghost triadic effects in relational event models. *Psychometrika,**89*(1), 151–171. 38446394 10.1007/s11336-024-09952-x

[30] Juozaitienė, R., & Wit, E. C. (2024b). Nodal heterogeneity can induce ghost triadic effects in relational event models. *Psychometrika,**89*(1), 151–171. 38446394 10.1007/s11336-024-09952-x

[31] Kozlowski, S. W. J., & Klein, K. J. (2000). A multilevel approach to theory and research in organizations: Contextual, temporal, and emergent processes. *Multilevel theory, research, and methods in organizations: Foundations, extensions, and new directions* (pp. 3–90). Jossey-Bass/Wiley.

[32] Krivitsky, P. N., & Handcock, M. S. (2014). A separable model for dynamic networks. *Journal of the Royal Statistical Society. Series B, Statistical Methodology,**76*(1), 29–46. 24443639 10.1111/rssb.12014 PMC3891677

[33] Lakdawala, R., Leenders, R., & Mulder, J. (2025). Not all bonds are created equal: Dyadic latent class models for relational event data. arxiv preprint. arXiv:2501.04418

[34] Lakdawala, R., Meijerink-Bosman, M., Leenders, R., & Mulder, J. (2020). *remulate: R package for simulating relational data*. Tilburg University.

[35] Leenders, R. T. A. J., Contractor, N. S., & DeChurch, L. A. (2016). Once upon a time: Understanding team processes as relational event networks. *Organizational Psychology Review,**6*(1), 92–115.

[36] Leonardelli, G. J., Pickett, C. L., & Brewer, M. B. (2010). Optimal distinctiveness theory. In *Advances in experimental social psychology* (Vol. 43, pp. 63–113). Academic Press.

[37] Lospinoso, J., & Snijders, T. A. (2019). Goodness of fit for stochastic actor-oriented models. *Methodological Innovations,**12*(3), 2059799119884282.

[38] Meijerink-Bosman, M., Leenders, R., & Mulder, J. (2022). Dynamic relational event modeling: Testing, exploring, and applying. *PLoS ONE,**17*(8), Article e0272309. 35913924 10.1371/journal.pone.0272309 PMC9342787

[39] Mulder, J., & Hoff, P. D. (2024). A latent variable approach for modeling relational data with multiple receivers. *The Annals of Applied Statistics,**18*(3), 2359–2381.

[40] Mulder, J., & Leenders, R. T. A. J. (2019). Modeling the evolution of interaction behavior in social networks: A dynamic relational event approach for real-time analysis. *Chaos, Solitons & Fractals,**119*, 73–85.

[41] Newman, M. E. J. (2006). Modularity and community structure in networks. *Proceedings of the National Academy of Sciences of the United States of America,**103*(23), 8577–8582. 16723398 10.1073/pnas.0601602103 PMC1482622

[42] Nicosia, V., Tang, J., Mascolo, C., Musolesi, M., Russo, G., & Latora, V. (2013). Graph metrics for temporal networks (pp. 15–40). arXiv:1306.0493 [physics]

[43] Perry, P. O., & Wolfe, P. J. (2013). Point process modelling for directed interaction networks. *Journal of the Royal Statistical Society: Series B (Statistical Methodology),**75*(5), 821–849.

[44] Schecter, A., & Quintane, E. (2020). The power, accuracy, and precision of the relational event model. *Organizational Research Methods,**24*, 802–829.

[45] Shafiee Kamalabad, M., Leenders, R., & Mulder, J. (2023). What is the point of change? Change point detection in relational event models. *Social Networks,**74*, 166–181.

[46] Snijders, T. A., van de Bunt, G. G., & Steglich, C. E. (2010). Introduction to stochastic actor-based models for network dynamics. *Social Networks,**32*(1), 44–60.

[47] Snijders, T. A. B., & Steglich, C. E. G. (2015). Representing micro–macro linkages by actor-based dynamic network models. *Sociological Methods & Research,**44*(2), 222–271. 25960578 10.1177/0049124113494573 PMC4422512

[48] Stadtfeld, C. (2014). *Events in social networks: A stochastic actor-oriented framework for dynamic event processes in social networks*. KIT Scientific Publishing.

[49] Stadtfeld, C., & Block, P. (2017). Interactions, actors, and time: Dynamic network actor models for relational events. *Sociological Science,**4*, 318–352.

[50] Valente, T. W. (2005). Network models and methods for studying the diffusion of innovations. In J. Scott, P. J. Carrington, & S. Wasserman (Eds.), *Models and methods in social network analysis, structural analysis in the social sciences* (pp. 98–116). Cambridge University Press.

[51] Valente, T. W. (2012). Network interventions. *Science,**337*(6090), 49–53. 22767921 10.1126/science.1217330

[52] Valente, T. W. (2017). Putting the network in network interventions. *Proceedings of the National Academy of Sciences,**114*(36), 9500–9501. 10.1073/pnas.1712473114 PMC5594703 28851836

[53] Wang, C., Butts, C. T., Hipp, J., & Lakon, C. M. (2020). Model adequacy checking/goodness-of-fit testing for behavior in joint dynamic network/behavior models, with an extension to two-mode networks. *Sociological Methods & Research,**51*, 1886–1919.

